# Sleep quality and neurocognitive functioning in metabolic syndrome

**DOI:** 10.4102/sajpsychiatry.v24i0.1296

**Published:** 2018-09-26

**Authors:** Sharain Suliman, Leigh L. van Den Heuvel, Sanja Kilian, Jonathan Carr, Robin Emsley, Soraya Seedat

**Affiliations:** 1 Department of Psychiatry, Faculty of Medicine and Health Sciences, Stellenbosch University, South Africa; 2 Department of Neurology, Faculty of Medicine and Health Sciences, Stellenbosch University, South Africa

## Abstract

**Background:**

The incidence of metabolic syndrome (MetS), a cluster of metabolic risk factors in a single individual, is increasing worldwide, making it important to study the possible risk and protective factors. Accumulating evidence has suggested sleep deprivation and/or fragmentation is among the key factors involved in the onset and treatment resistance of MetS components. Moreover, bidirectional associations between sleep complaints and MetS have been described. In addition, there is mounting evidence of the effect of MetS on cognitive functioning.

**Aims:**

The aim of this study was to assess whether MetS and sleep complaints are associated with clinically determined neurocognitive disturbances in a sample of participants with MetS symptoms, ranging from none to all criteria met.

**Methods:**

Participants comprised 153 mixed race individuals from the Western Cape province of South Africa. Sleep (Pittsburgh Sleep Quality Index), neurocognition (Repeatable Battery for the Assessment of Neuropsychological Status) and anthropometric (MetS components) assessments were performed on all participants. A hierarchical regression model, including potentially confounding variables (e.g. IQ), demographic variables (e.g. age and gender) and clinical variables (e.g. [BMI] and cholesterol), was then constructed.

**Results:**

The model was significant: adjusted *R* square = 0.486; *F*(13, 110) = 9.952, *p* < 0.0001. The demographic variables accounted for 32.3% of variability. This increased to 48.5% when the clinical variables were added. Sleep and metabolic criteria only added 0.1%.

**Discussion:**

Although we did not find sleep and metabolic factors to significantly influence cognition when other factors were accounted for, further investigation into risk and outcome factors, such as these, may assist in the identification of mechanistic links, which may also improve management of patients who are at risk, thereby improving health outcomes.

